# Case Report: III° atrioventricular block due to fulminant myocarditis managed with non-invasive transcutaneous pacing

**DOI:** 10.12688/f1000research.14000.2

**Published:** 2018-03-23

**Authors:** Kiran Devkota, Ya Hong Wang, Meng Yi Liu, Yan Li, You Wei Zhang

**Affiliations:** 1 Department of Pediatrics I, Renmin Hospital, Hubei University of Medicine, Hubei, China

**Keywords:** III° A-V block, Fulminant Myocarditis, non-invasive transcutaneous cardiac pacing, ECMO

## Abstract

Fulminant myocarditis is a life-threatening clinical condition. It is the inflammation of myocardium leading to acute heart failure, cardiogenic shock and cardiac arrhythmias. Incidence of fulminant myocarditis is low and mortality is high. Most grievous complications of fulminant myocarditis is mainly cardiac arrhythmias; if there is delay on active management of the patient, it may be fatal. Here, we describe a case of III° atrioventricular block due to fulminant myocarditis that was managed with non-invasive transcutaneous cardiac pacing in the absence of ECMO. The non-invasive transcutaneous pacemaker is a safe, effective and convenient device to revert arrhythmias.

## Abbreviations

A-V block- Atrioventricular block; ECG- Electrocardiogram; ECMO- Extracorporeal Membrane Oxygenation; LVEF- Left ventricular ejection fraction

## Introduction

Fulminant myocarditis is a life-threatening clinical condition. It is inflammation of myocardium leading to acute heart failure and cardiac conduction abnormalities with rapid deterioration. There are about 10–38% cases of fulminant myocarditis among all cases of acute myocarditis ^1^. Causes of fulminant myocarditis may be of viral, bacterial or non-infectious origin ^1– 3^. Diagnosis of fulminant myocarditis is very difficult because of non-specific symptoms and diagnostic tools. There may be signs of acute heart failure, cardiogenic shock, or life-threatening cardiac arrhythmias. Cardiac arrhythmias are varied in presentation, ranging from Sinus arrest, AV block, Ventricular tachycardia and Ventricular fibrillation during acute phase ^4^. Here we present a case of fulminant myocarditis presenting with different clinical features and IIIº A-V block, which was successfully managed with non-invasive transcutaneous pacing.

## Case report

**Figure 1.**
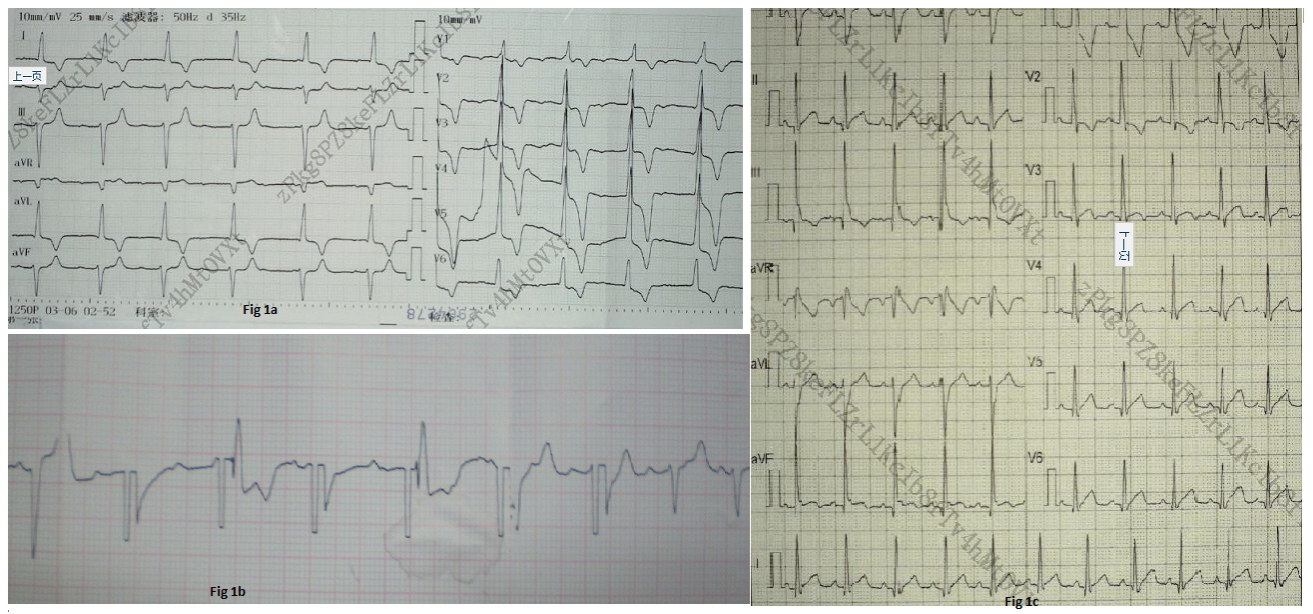
( **A**) Electrocardiogram (ECG) at emergency showing- III°atrio-ventricular block; left anterior fascicular block, ST-T changes; ( **B**) ECG recording during transcutaneous pacing; ( **C**) ECG at the time of discharge, which is normal.

A 3 ½ year old female child having a productive cough and 5–6 episodes of the passage of loose stool for 2 days was taken to local hospital after she had sudden convulsion for about 2 minutes. At the hospital, she again had convulsion for 1–2 minutes and her heart rate dropped to 30 bpm. She was given atropine, dexamethasone, dextrose and per rectal choral hydrate at the local hospital (doses not known) and immediately referred to Renmin Hospital. She had no significant past medical history, drug sensitivity or allergies. On arrival she was conscious but lethargic and dyspnoeic. Both pupils were round, 4mm and reactive to light. Her vitals were T36.4 °C, HR 62 bpm, RR 65/min, BP 90/41mmHg and SPO2 80%. Table 1 shows the results of routine laboratory measurements. Her lips were cyanosed and she had pale and cold extremities. Neck and throat examination was normal. Chest examination showed b/l crackles and irregular heart rate with no murmurs. The abdomen was soft and non-tender. Electrocardiogram (ECG) showed- III° atrio-ventricular (A-V) block; left anterior fascicular block, ST-T changes ( Figure 1). She had cardiac arrest three times in the emergency department at 15–20 minutes interval. She was resuscitated with chest compression along with Isoproterenol and adrenaline. Subsequently, her heart rate was maintained in between 70–90 beats/min. Provisional diagnosis was acute fulminant myocarditis with bronchial pneumonia.

**Table 1.** Laboratory investigations from day of admission to discharge.

Blood Investigations	0 DOA		1 DOA		2 DOA		3 DOA		4 DOA		7 DOA		12 DOA		19 DOA		27 DOA
WBC (4–10×10^9/L)	19.86	↑	20.02		17.16	↑	18.42	↑	15.61	↑	12.51	↑	10.52	↑	10.01	↑	8.43
Neutrophils (50–75%)	68.7		70.8		85.4	↑	80.47	↑	47.6	↓	55.8		69.3		57.6		57.8
Lymphocytes (20–40%)	23.9		22.4		10.4	↓	14.4	↓	45.9	↑	37		27		35.9		35
Monocytes (3–8%)	7.3		6.2		4.1		4.1		5.6		5		3.1		5.6		5
Eosinophils (0.5–5%)	0	↓	0.5		0	↓	0	↓	0.6		1.6		0.4	↓	0.6		1.6
Basophils (0–1%)	0.1		0.1		0.1		0.1		0.3		0.6		0.2		0.3		0.6
ANC (2–7.5×10^9/L)	13.65	↑	14.1		14.67	↑	13.67	↑	7.42		3.89		7.83	↑	4.42		3.89
ALC (0.8–4×10^9/L)	4.74	↑	2.22		1.78		1.88		7.16	↑	5.86	↑	1.68		3.16		2.86
Hemoglobin (110–170 g/L)	110	↓	107		95	↓	97	↓	108	↓	110	↓	116		122		129
Platelets count(100– 300×10^9/L)	203		200		143		132		403	↑	257		311	↑	403	↑	257
ESR (0–15 mm in 1 hr)	1																
Blood Glucose (3.89–6.11 mmol/L)	11.2	↑	6.1		3.8	↓			4.6		4.2				7.2	↑	5.3
Potassium (K) (3.5–5.4 mmol/L)	4.3	↓	5.18		4.46				3.5		3.52				4.13		4.4
Sodium (Na) (135–148 mmol/L)	131	↓	138		129	↓			133.8	↓	134.6	↓			141		142
Calcium (Ca) (2.05–2.55 mmol/L)	1.58	↓	1.6	↓	1.62	↓			2.27		2.23				2.38		
Blood Urea (1.8–7.1 mmol/L)	21.35	↑	21.45	↑	21.79	↑			6.88		4.98				5.29		4.1
creatinine (44–106 umol/L)	158.5	↑	167	↑	211.6	↑			63.5		57.5				49.6		50
Uric Acid (129–417 umol/L)	977	↑	980	↑	935	↑			274		253				169		
ALT (8–40 U/L)	8526	↑	6589	↑	1406	↑			552	↑	52	↑			27		
AST (5–40 U/L)	4724	↑	3245	↑	2319	↑			309	↑	309	↑			31		
α HBDH ( 72–182 IU/L)	3895	↑	2145	↑	1061	↑			631	↑	489	↑			192	↑	
ALP (40–150 IU/L)	151	↑	144		140	↑			127		126				141		
γGGT (7–54 U/L)	26		28		28				58	↑	53				48		
LDH (100–300 IU/L)	10140	↑	9876	↑	2155	↑			746	↑	456	↑			223		145
Total Protein (60–85 g/L)	56.8	↓	60.4		63.6				66.8		72.8				72.7		
Albumin (35–55 g/L)	33.5	↓	33	↓	32	↓			39		43				41.1		
Globulin ( 20–35 g/L)	23.3		26.9		31.6				34		34				31.6		
Creatine Kinase (25–200 IU/L)	1170	↑	1245	↑	1679	↑			109		89				39		35
CKMB (0–25 U/L)	247	↑	187	↑	104	↑			48	↑	48	↑			10		13
Troponin T (0–0.08 ng/ml)	0.361	↑													0.024		0.018
ASO Titre (0–166 IU/ml)	7														24		
CRP (0–10 mg/L)	0.9												0.1				
**Atrial Blood Gas**																	
PH (7.35 – 7.45)	7.34	↓	7.39		ANC: Absolute Neutrophil count ALC: Absolute Lymphocytes cont													
PaO2 (80–100 mmHg)	82.1		88															
PaCO2 (35–45 mmHg)	23	↓	36															
HCO3 (22–26 mEq/L)	13.3	↓	24.2															
Anion Gap (10–15 mEq/L)	31	↑	15.2															
**Stool Routine**	Normal																	
**Urine routine**	Normal																	
**Mycoplasma titer**	Negative																	

The patient was admitted to hospital after explaining the disease condition and prognosis to parents. She was on continuous oxygen, dopamine, diazepam, Immunoglobulin, ceftriaxone, IV fluids on a maintenance dose and nebulization with Ipratropium bromide (250mcg/ nebulization) along with a high dose of vitamin C (to reduces the risk of myocardial injury), Coenzyme Q 10 (for myocardial protection), fructose diphosphate (to improve cardiac metabolism) and mannitol (to reduce cerebral edema). However, she again had a cardiac arrest. In addition, ECG showed sinus P wave and no QRS with heart rate dropped from 70 to 20bpm. The patient was resuscitated with chest compression, atropine, and adrenaline. Isoproterenol was started at 1.5mcg/kg/min and increased up to 2mcg/kg/min. Subsequently, her heart rate was maintained at 60–70 bpm. Her heart rate again decreased to 30bpm when isoproterenol was discontinued. As there was no extracorporeal membrane oxygenation (ECMO) machine in our hospital and transfer was not possible, the patient was prepped for non-invasive transcutaneous cardiac pacing.

Cardiac pacing was adjusted to 16 mA and rate 90 bpm. The patient’s heart rate was controlled at 80–100 bpm. Her complexion gradually became reddish, cyanosis gradually improved but she had developed eyelid edema. She had passed urine about 130ml twice in 12 hours. Dopamine was increased to 7mcg/kg/min and she was started on furosemide. 

**Figure 2.**
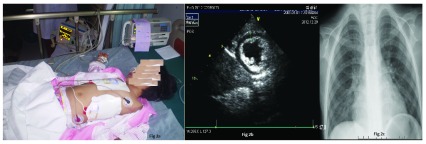
( **A**) Patient on non-invasive transcutaneous pacing; ( **B**) Echocardiography after 48 hours of admission showing left ventricular myocardial wall thickening and thickening of endocardium; ( **C**) Chest X ray showing increased lung texture and enlarged cardiac shadow.

At first 24 hours after cardiac pacing, the patient was conscious. She had passed urine 4 times about 700ml. But facial puffiness was still present. Her heart rate was maintained at the rate of 110–130bpm and SPO2 was 96% with oxygen. Chest pacing was reduced to 14 mA, and the frequency was changed to 70 bpm. After 48hours of pacing, the heart rate was improved to 100–110bpm with few ventricular premature beats. Then pacing was reduced to 12 mA, frequency changed to 60bpm. The pacing current and frequency were gradually slowed down and discontinued. Then, sinus rhythm was established with the heart rate of 100–110bpm with ECG monitoring. The heart rate fluctuated at 80–100bpm with frequent ventricular premature beats. Echocardiography showed left ventricular myocardial wall thickening and thickening of endocardium with left ventricular ejection fraction (LVEF 50%), suggestive of endocardial fibroelastosis (EF). Chest radiograph showed increased lung texture and enlarged cardiac shadow ( Figure 2). Captopril, hydrochlorothiazide, and spironolactone were started to reduce cardiac remodeling and to protect heart function. Furosemide was continued. Mannitol was stopped after the patient’s MRI scan revealed normal findings.

The patient’s HR was in between 80–100 bpm, with blood pressure was increasing gradually. Dopamine was tapered and stopped at 72 hours, after her BP reached 110/78 mmHg. The chest became gradually clear and her heart sounds were also normal. ECG monitoring also showed improvement with decreased numbers of premature beats and gradual change of S-T segments towards normal.

The patient was discharged on the 28th day after admission after her all routine investigations returned to normal ( Table 1). ECG showed sinus rhythm with heart rate 102 bpm. Echocardiography showed normal cardiac chambers, normal wall motion with EF 60%. Her final diagnosis was fulminant myocarditis with III° A-V block and bronchial pneumonia. On discharge, the patient was advised to continue captopril 6.25mg bid, metoprolol succinate 6.25mg bid, prednisone 1mg / kg orally for six months.

 At six month follow up the patient’s echocardiography had returned to normal with LVEF 65%, and prednisone was reduced to 0.5mg/kg orally for 15 days and with a tapering dose for the next 15 days. After one year follow-up, she had no complaints and no significant abnormalities noted on echocardiography.

All doses of medications can be seen in Table 2.

**Table 2.** List of medications, including doses and duration, given to the patient during hospital admission.

Doses of medicine and duration		
Medicine	Doses	Route/duration
Atropine	0.25mg	IV When reqired
Adrenaline	0.2mg	IV When reqired
Isoproterenol	0.2mcg	IV- bolus at ER
	0.15mcg/kg/min	IV in 50ml of 5% glucose
	0.2mcg/kg/min	IV in 50ml of 5% glucose
Dopamine	3–5mcg/kg/min	IV in 50ml of 5% glucose
Diazepam	0.5mg/kg	IV when reqired
Phenobarbital	2mg/kg	IV when reqired
Mannitol	42 ml	IV 6 hourly for 2 days from DOA
	42 ml	IV 8 hourly for next 2 days
	42ml	IV 12 hourly for next 2 days then stop
Fructose diphosphate	3.4g /OD	IV for 10 days
Ceftriaxone	100mg/kg/day	IV 12 hourly from DOA for 10 days
Piperacillin tazobactam	1.125gm/day	IV 12 hourly from 3 DOA for 10 days
Immunoglobulin	5 gm	IV daily for 5 days
Methylprednisolone	1.5mg/kg/day	IV for 5 days
prednisone	10 mg / OD	PO from 6 DOA and on discharge also
Captopril	6.25mg / BID	PO from 3 DOA and on discharge also
Spironolactone	10mg/OD	PO from 5 DOA and on discharge also
Furosemide	10mg	IV 12hourly from 2nd DOA to 5DOA
Hydrochlorothiazide	10mg	PO 12 hourly from 5 DOA till discharge
Coenzyme Q10	5 mg	PO 8hourly from 2nd DOA till discharge
Vitamin C	3 gm	IV 12 hourly from 2nd DOA till discharge

## Discussion

In children, sometimes myocarditis is self-limiting. However, if it progresses there is the risk of acute cardiac failure, hemodynamic disturbances, and arrhythmias leading to significant morbidity and mortality. Mortality due to myocarditis for infants is more than 75%, whereas for children it is more than 25% ^1, 5– 9^. There is no any specific clinical course and investigations to diagnose fulminant myocarditis. Initially, they present with flu-like symptoms and later develop sudden onset of cardiac symptoms that rapidly deteriorate ^2^. Neonates may present with fever, poor feeding, and listlessness and sometimes with danger signs like apnea, episodic cyanosis, and diaphoresis. Older children present with respiratory or gastrointestinal symptoms. Among them only a few present with chest pain ^10^. Diagnosis is mainly done on the basis of: Clinical presentation, blood profile, including CBC, electrolytes, creatinine kinase, creatine kinase MB isoenzyme, C-reactive protein, Troponin T, Troponin I, antistreptolysin O titer, polymerase chain reaction to detect viral antigens, autoantibodies marker, liver enzymes, ECG, Echocardiography, ultrasonography and even Cardiac MRI ^3^ which are mostly supportive. If echocardiography shows low LVEF in children with fulminant myocarditis, the prognosis is poor ^11^. Mortality in fulminant myocarditis is mainly due to cardiac arrhythmias among which structural changes, parameters of ventricular dynamics and vascular changes are responsible for the increased incidence ^12^. Acute fulminant myocarditis, if properly and aggressively treated, has excellent long-term survival even if the patient may present with severe hemodynamic compromise ^13^. Complete heart block on initial ECG may also have an excellent prognosis, although mechanical assistance may be warranted as shown in the study by Lee E Y *et al.* ^1^. This can be managed with percutaneous cardiopulmonary support, ECMO, intra-aortic balloon pumping, or the ventricular assisted device ^14, 15^. ECMO remains an effective approach in children for the management of acute fulminant myocarditis ^16^. In addition, intravenous immunoglobulin and high dose steroids help to reduce inflammation ^17^. Patients managed with immunoglobulin, steroids or mechanical support for fulminant myocarditis may have higher survival rate compared to those not receiving these therapies ^14^. 

In the present case, we tried to manage initially with Isoproterenol but were unsuccessful. So we applied the non-invasive transcutaneous pacemaker to revert the A-V block. Pads or electrodes detachment, patient non-cooperation and skin-burn due to high voltage electric current are its limitations. In contrast, intraventricular cardiac pacing is time-consuming; much more risky and surgical site wound infection is common. Beland *et al*, Kelly *et al* in their articles note that non-invasive transcutaneous pacemaker is the safe, effective and suitable equipment for children ^18, 19^.

## Conclusion

Acute fulminant myocarditis is a grievous condition with high morbidity and mortality. No delay should be had on starting immunoglobulin and steroids if suspected. If there is the arrhythmia, the patient should be immediately started on ECMO, percutaneous cardiopulmonary support or ventricular assisted device. If these are not available, then non-invasive transcutaneous cardiac pacing must be started, which is a safe, convenient and cost-effective device to revert arrhythmias caused by myocarditis.

## Consent

We have taken written informed consent from the child`s legal guardian (her father) to use and publish his child`s medical case history and any accompanying images.

## Data availability

*All data underlying the results are available as part of the article and no additional source data are required.*
