# Supplementary material for: Case Report: III° atrioventricular block due to fulminant myocarditis managed with non-invasive transcutaneous pacing
Source: F1000Res. 2018 Mar 23;7:239. Originally published 2018 Feb 28. [Version 2] doi: 10.12688/f1000research.14000.2 (PMC5871802; doi:10.12688/f1000research.14000.2)
Supplement: Peer review history

# Peer review: Referee response for version 2

**DOI:** 10.5256/f1000research.15664.r32382

**Published:** 2018-03-26

**Authors:** Sabine Pankuweit

**Affiliations:** 1 Department of Cardiology, Angiology and Intensive Care, Philipps University of Marburg, Marburg, Germany

Authors have answered the questions raised by the reviewer in an appropriate way

I have read this submission. I believe that I have an appropriate level of expertise to confirm that it is of an acceptable scientific standard.

# Peer review: Referee response for version 2

**DOI:** 10.5256/f1000research.15664.r32381

**Published:** 2018-03-26

**Authors:** Mani Prasad Gautam

**Affiliations:** 1 Department of Medicine, Bharatpur Hospital, Chitwan, Nepal

The Authors have discussed an interesting case of fulminant myocarditis in a child which have been managed with transcutaneous pacing for the third degree AV block along with other supportive care including intravenous methylprednisolone and immunoglobulin. In overall the article is at satisfactory level in its scientific contents and usefulness to other practitioners. Some improvement has been seen after the first review and have adequate standard of scientific publication.

I have read this submission. I believe that I have an appropriate level of expertise to confirm that it is of an acceptable scientific standard.

# Peer review: Referee response for version 1

**DOI:** 10.5256/f1000research.15216.r31964

**Published:** 2018-03-14

**Authors:** Sabine Pankuweit

**Affiliations:** 1 Department of Cardiology, Angiology and Intensive Care, Philipps University of Marburg, Marburg, Germany

The authors described a case of III° atrioventricular block due to fulminant myocarditis that was managed with non-invasive transcutaneous cardiac pacing in the absence of ECMO. In this case the non-invasive transcutaneous pacemaker was a safe, effective and convenient device to revert arrhythmias.

 Nevertheless, as the authors stated, if there is the arrhythmia, the patient should be immediately started on ECMO, percutaneous cardiopulmonary support or ventricular assisted device.

 Authors should specify and discuss, what the most important arrythmias with negative prognosis to what extend are.

 Are there any efforts made in this case, to get information on the etiology of the disease (e.g. viral, autoimmune), was there any discussion on an endomyocardial biopsy. 

 Data in children with regard to the prognosis and outcome in fulminant myocarditis should be added.

I have read this submission. I believe that I have an appropriate level of expertise to confirm that it is of an acceptable scientific standard.

# Peer review: Referee response for version 1

**DOI:** 10.5256/f1000research.15216.r31290

**Published:** 2018-03-05

**Authors:** Mani Prasad Gautam

**Affiliations:** 1 Department of Medicine, Bharatpur Hospital, Chitwan, Nepal

The Authors have discussed an interesting  case  of fulminant myocarditis in a child which have been managed with transcutaneous pacing for the third degree AV block along with other supportive care including intravenous methylprednisolone and immunoglobulin. In overall the article is at satisfactory level in its scientific contents and usefulness to other practitioners. But there is some space to improve its rating. The background information provided here is an overall information, it would have been better if the author has also focused on various arrhythmias associated with fulminant myocarditis as the myocarditis tends to have various refractory arrhythmias including ventricular tachycardia. ^1^ It would have been better if the authors were focused on particular theme, such as on various arrhythmias and their medical management or overall medical management. The case description is somehow lacking smoothness in the flow of information. That could be because lack of cohesion in sentences and writing in the form of clinical vignette can improve its content. Similarly there are a lot of English grammar related errors and rampant use of short form that should be corrected. In addition, it is advised to use scientific words rather than layman terms in academic writings. Taking help from an English language expert might also improve its contents. The case report section should be made little bit short. The discussion section should be more descriptive by discussing clinical features of myocarditis including various arrhythmias and medical management of myocarditis. The use of Vitamin C, Coenzyme Q10, Fructose diphosphate and Mannitol can not be understood from the discussion section which I was expecting there. I hope, the revision after considering above advice will improve the scientific content of the article.

I have read this submission. I believe that I have an appropriate level of expertise to confirm that it is of an acceptable scientific standard, however I have significant reservations, as outlined above.

## References

[1] GautamMSogunuruGSubramanyamGViswanathR: Tuberculous myocarditis presenting as a refractory ventricular tachycardia of biventricular origin. *Journal of College of Medical Sciences-Nepal*.2012;7(2) : 10.3126/jcmsn.v7i2.6686 10.3126/jcmsn.v7i2.6686
